# Drug fever induced by Xingnaojing injection: A case report

**DOI:** 10.1097/MD.0000000000048482

**Published:** 2026-05-01

**Authors:** Weiqian Liu, Bo Gao, Shaowei Xie, Ye Yuan, Yu Yin

**Affiliations:** a Department of Rehabilitation Medicine, Hebei General Hospital, Shijiazhuang, Hebei, China; b Hebei Provincial Key Laboratory of Cerebral Networks and Cognitive Disorders, Hebei General Hospital, Shijiazhuang, Hebei, China; c Department of Urinary Surgery, Hebei General Hospital, Shijiazhuang, Hebei, China.

**Keywords:** adverse drug reactions, case report, drug fever, postoperative management following cerebral hemorrhage, Xingnaojing injection

## Abstract

**Rationale:**

The application of Xingnaojing injection is widespread; however, drug-induced fever associated with this medication is challenging to diagnose. Previous literature rarely reports cases of drug-induced fever resulting from the use of Xingnaojing injection.

**Patient concerns:**

A 46-year-old male patient, who underwent surgery for cerebral hemorrhage, presented with fever. The condition is considered to be drug fever induced by the administration of Xingnaojing.

**Diagnoses:**

Considering the potential for drug fever associated with the administration of Xingnaojing injection.

**Interventions:**

Discontinue the use of Xingnaojing injection.

**Outcomes:**

The patient has shown improvement and is being discharged.

**Lessons:**

Clinical practitioners should enhance the prevention and management of adverse drug reactions to reduce both patient hospitalization duration and economic costs, thereby ensuring the safety of medication use for patients.

## 1. Introduction

The Xingnaojing injection is a traditional Chinese medicinal compound preparation made from ingredients such as musk, curcuma, borneol, gardenia fruit, polysorbate 80, and sodium chloride. It possesses various therapeutic effects including heat-clearing and detoxifying properties, cooling the blood and promoting circulation, as well as opening the orifices and awakening the mind. Clinically, it is widely used for conditions such as acute poisoning, cerebrovascular diseases, coronary heart disease, cranial trauma, encephalitis, acute infectious fever in children, heatstroke, pulmonary-brain syndrome from respiratory failure among other ailments. However, with its extensive application has come an increasing number of reports concerning adverse drug reactions (ADRs) associated with Xingnaojing injection.^[1-3]^ Herein we report a case of drug fever induced by Xingnaojing injection.

## 2. Case presentation

The patient is a 46-year-old male, who was admitted on April 28, 2021, at 16:04 due to limited mobility of the left limbs for over one month. The diagnoses are as follows: hemiplegia; postoperative status after cerebral hemorrhage; stage II hypertension (very high risk). Upon admission, treatment with Xingnaojing and Piracetam Sodium Chloride injection via intravenous infusion was initiated. The patient began to experience fever from May 8, without chills or rigor, and exhibited no significant accompanying symptoms. The patient was first tested on May 8, revealing a mild decrease in white blood cell count (4.68 × 10^9^/L) and a slight increase in C-reactive protein (8.72 mg/L). A follow-up examination on May 13 indicated a significant elevation in inflammatory markers (C-reactive protein 28.12 mg/L, procalcitonin 0.111 ng/mL, erythrocyte sedimentation rate 37 mm/h), along with a further decline in white blood cell count (2.71 × 10^9^/L) and an increase in serum ferritin levels (537.5 ng/mL). Immunological tests showed positive results for antinuclear antibody at a titer of 1:3200 (nuclear speckled pattern), with notable elevations in anti-Ro-52, anti-Sjögren’s-syndrome-related antigen A, and anti-Sjögren’s-syndrome-related antigen B antibodies; Immunoglobulin G levels were also elevated (30.68 g/L; Table 1). The remaining test results can be found in the supplementary materials (Table S1, Supplemental Digital Content, https://links.lww.com/MD/R770). After discontinuing all intravenous medications on May 14, the patient’s temperature returned to normal within 3 days. On May 18, the patient was transferred to Traditional Chinese Medicine department for continued treatment, where they received infusions of XINAOJING and Piracetam Sodium Chloride injection. The patient experienced a recurrence of high fever; however, upon discontinuation of XINAOJING on May 19, their body temperature normalized again. It is suspected that the patient’s drug-induced fever was caused by the administration of XINAOJING injection. Subsequently, the patient improved and was discharged from hospital.

**Table 1:** Key laboratory and imaging findings at different time points.

Date (hospital day)	Hematology/inflammatory markers	Immunology (positive findings)	Imaging/other findings
May 8 (D1)	WBC: 4.68 × 10^9^/L ↓; CRP: 8.72 mg/L ↑	–	Cranial CT: postoperative changes; Chest CT: fibrous stripes in RLL, dependent atelectasis in both lower lobes; fatty liver
May 13 (D6)	WBC: 2.71 × 10^9^/L ↓; CRP: 28.12 mg/L ↑; PCT: 0.111 ng/mL ↑; ESR: 37 mm/h ↑; Ferritin: 537.5 ng/mL ↑	ANA: 1:3200 (speckled); anti-Ro-52: 126 ↑; anti-SSA: 117 ↑; anti-SSB: 101 ↑; IgG: 30.68 g/L ↑	–

ANA = antinuclear antibody, Anti-SSA = Anti-Sjögren’s-syndrome-related antigen A (also called Anti-Ro antibody), Anti-SSB = Anti-Sjögren’s-syndrome-related antigen B (also called Anti-La antibody), CRP = C-reactive Protein, ESR = erythrocyte sedimentation rate, IgG = immunoglobulin G, PCT = procalcitonin, WBC = white blood cell (count).

## 3. Discussion

Drug fever refers to the fever that may occur in patients after the administration of 1 or more medications, which is not associated with the underlying disease or infection. Typically, body temperature returns to normal upon discontinuation of the offending drug, making it a common type of drug-induced illness. One of the main characteristics of drug fever is that symptoms usually resolve within 1 to 3 days after cessation of the related medication.^[4]^ The diagnosis of drug fever is primarily exclusionary; it requires ruling out other diseases or infections as potential causes of fever while considering a comprehensive assessment that includes patient symptoms, signs, inflammatory markers, and imaging results. This is particularly important for patients who have been prescribed multiple antimicrobial agents and/or non-antimicrobial medications – if they experience persistent elevated temperatures without any allergic reactions, careful evaluation must be conducted to exclude postoperative infections or exacerbation of preexisting conditions.

The diagnosis of drug-induced fever lacks specific criteria. When a patient presents with fever that cannot be attributed to common causes such as infection, trauma or surgery, tumors, or rheumatic diseases, the possibility of drug-induced fever must be considered. In such cases, it is essential to undertake a comprehensive analysis that includes the patient’s medication history, clinical manifestations (symptoms and signs), laboratory tests, cessation of suspected pyretogenic drugs, and rechallenge testing. These factors collaboratively aid in assessing the temporal relationship between 1 or more drugs and the onset of fever.

According to the Naranjo scale (Table 2), which evaluates the probability of ADRs related to medication, the algorithm achieved a score of 7, indicating a high likelihood of correlation. Components such as musk, gardenia fruit, and borneol contain macromolecular proteins and fatty acids that can act as antigens upon entering the body, triggering a series of immune responses. This may subsequently lead to clinical manifestations affecting skin mucosa and respiratory systems.^[5]^ The excipient in Xingnaojing injection includes Polysorbate 80, one of the most commonly used solubilizing agents. Polysorbate 80 enhances the solubility of poorly soluble components in traditional Chinese medicine, thereby improving the stability of injections. However, some studies have indicated that polysorbates possess certain sensitizing properties,^[6]^ which could be 1 reason for the occurrence of ADRs associated with Xingnaojing injection. In a total of 62 reported cases^[7]^ involving ADRs related to Xingnaojing injection, most instances demonstrated 2 or more clinical manifestations across multiple systems and organs – with damage to skin and its appendages being most prevalent. This was followed by gastrointestinal damage, systemic injury, respiratory system impairment, as well as central and peripheral nervous system disorders. Among 249 cases^[8]^ linked to Xingnaojing injection ADRs primarily involving injurious effects on skin and its attachments as well as systemic harm – as well as central and peripheral nervous system complications – the main clinical symptoms consisted of rashes; dizziness; headaches; palpitations; and chest tightness – along with 7 reported cases exhibiting anaphylactic shock associated with systemic damage. Over past reports regarding ADR occurrences are largely concentrated within 30 minutes post-medication administration – predominantly presenting as hypersensitivity reactions.^[9,10]^ In this particular case occurring ten days after medication administration when determining ADRs was not initially prioritized.

**Table 2:** Naranjo ADR evaluation scale.

Related questions			
	Score		
Related questions	Yes	No	Unknown
1. Is there any conclusive report before this ADR?	1√	0	0
2. Do the ADR occur after the use of suspect drugs?	2√	−1	0
3. Do the ADR relieve after drug withdrawal or use of antagonist?	1√	0	0
4. Does the ADR recur after reuse of the suspect drug?	2√	−1	0
5. Are there other reasons that can cause the ADR independently?	−1	2	0
6. Does the ADR repeat after the application of placebo?	−1	1	0
7. Does the drug reach toxic concentration in blood or other body fluids?	1	0	0
8. Is the ADR aggravated (relieved) with the increase (decrease) of dose?	1√	0	0
9. Has the patient ever been exposed to the same or similar drugs and had similar reactions	1	0	0
10. Is there any objective evidence to confirm the reaction?	1	0	0
Total score			

The total score ≥ 9 shows that the causal relationship of adverse drug reactions is definite; the total score 5 to 8 is probably or likely to be relevant; the total score 1 to 4 is possible to be relevant; the total score ≤ 0 is doubtful to be relevant.

ADR = adverse drug reactions.

In summary, drug fever lacks specific diagnostic criteria, often leading to underreporting and misdiagnosis in clinical practice, thereby increasing the risk of ADRs. Consequently, the recognition, diagnosis, and management of drug fever hold significant importance for rational medication use in clinical settings. During treatment, clinicians should enhance their efforts in the prevention and management of ADRs to reduce patients’ hospital stays and associated costs while ensuring safe medication practices for patients.

## Author contributions

**Conceptualization:** Weiqian Liu, Yu Yin.

**Investigation:** Weiqian Liu, Yu Yin.

**Methodology:** Weiqian Liu, Yu Yin.

**Resources:** Weiqian Liu.

**Writing – original draft:** Weiqian Liu.

**Writing – review & editing:** Bo Gao, Shaowei Xie, Ye Yuan.

## Supplementary Material
