# Supplementary material for: Drug fever induced by Xingnaojing injection: A case report
Source: Medicine (Baltimore). 2026 May 1;105(18):e48482. doi: 10.1097/MD.0000000000048482 (PMC13138454; doi:10.1097/MD.0000000000048482)
Supplement: Supplementary file 1 [file medi-105-e48482-s001.pdf]

Table S1. Additional laboratory and imaging findings (non-significant or negative results)

Date (Hospital day)	Category	Test/Parameter	Result	Reference range	Interpretation
5/8 (D1)	Urinalysis	Routine urine test	Within normal limits	—	Negative
5/8 (D1)	Ultrasound	Urinary system	Prostate: mild hyperplasia; left renal small cyst (suspected); bladder/ureters: no abnormality	—	Non-specific
5/13 (D6)	Microbiology	Urine culture	No bacterial growth (2 days)	—	Negative
5/13 (D6)	Microbiology	Blood culture	No bacterial growth (5 days, aerobic)	—	Negative
5/13 (D6)	Immunology	ANCA (p-ANCA, c-ANCA, MPO, PR3)	All negative	—	Negative
5/13 (D6)	Immunology	Anti-cardiolipin Ab (IgA/IgG/IgM)	2.4 RU/ml (within normal)	<20 RU/ml	Negative
5/13 (D6)	Immunology	Anti-β2 glycoprotein I Ab (IgA/IgG/IgM)	10.7 RU/ml (within normal)	<20 RU/ml	Negative
5/13 (D6)	Coagulation	Lupus anticoagulant (screen/confirm/NLR)	1.09 / 1.04 / 1.05	<1.2	Negative
5/13 (D6)	Immunoglobulins	IgM	0.97 g/L	0.4–2.3 g/L	Normal
5/13 (D6)	Complement	C3, C1q	Within normal range	—	Normal
5/13 (D6)	Tuberculosis	T-SPOT (A/B panels)	0 SFCs/2.5×10^5 PBMC	—	Negative
5/13 (D6)	Viral serology	HSV-IgG, CMV-IgG	Elevated, consistent with past infection	—	Clinically irrelevant
